# Expression of immunohistochemical markers in non-oropharyngeal head and neck squamous cell carcinoma in Ghana

**DOI:** 10.1371/journal.pone.0202790

**Published:** 2018-08-23

**Authors:** Osei Owusu-Afriyie, W. K. B. A. Owiredu, Kwabena Owusu-Danquah, Rita Larsen-Reindorf, Peter Donkor, Emmanuel Acheampong, Solomon E. Quayson

**Affiliations:** 1 Department of Molecular Medicine, School of Medical Science, Kwame Nkrumah University of Science and Technology, Kumasi, Ghana; 2 Department of Pathology, School of Medical Sciences Komfo Anokye Teaching Hospital, Kumasi, Ghana; 3 Department of Medical laboratory technology, Faculty of Allied Health, Kwame Nkrumah University of Science and Technology, Kumasi, Ghana; 4 Directorate of Dental, Eye, Ear, Nose & Throat, Komfo Anokye Teaching Hospital, Kumasi, Ghana; 5 Department of Maxillofacial Surgery, Dental School, KNUST, Kumasi, Ghana; 6 Department of Pathology, University of Ghana Medical School, Accra, Ghana; University of South Alabama Mitchell Cancer Institute, UNITED STATES

## Abstract

**Background:**

Head and neck cancers include carcinomas of the oral cavity, larynx, sinonasal tract and nasopharynx. Studies on molecular expression of prognostic tumour markers in Ghana are scarce. The purpose of this study was to determine the expression of p53, p16, EGFR, Cyclin-D1 and HER2 among patients with non-oropharyngeal head and neck squamous cell carcinoma (HNSCC).

**Methodology:**

Tissue microarrays from 154 histologically confirmed non-oropharyngeal HNSCC at the Komfo Anokye Teaching Hospital from 2006–2014 were constructed using duplicate cores of representative and viable areas from tumours. Expression of EGFR, p53, p16, Cyclin-D1 and HER2 was evaluated using immunohistochemistry.

**Results:**

For non-oropharyngeal HNSCC, majority of the cases (66.2%; 102/154) had stage IV disease. EGFR was the most expressed molecular marker (29.4%; 25/85) followed by p53 (24.0%; 29/121), p16 (18.3%; 23/126) and Cyclin-D1 (10.0%; 12/120). HER2 was not expressed in any of the cases. There was a significantly (p = 0.022) higher expression of Cyclin-D1 in tumours of the oral cavity (19.6%; 9/46) than in those of the larynx (4.7%; 2/43) and nose (3.2%; 1/31). Tumours in stages I–III were more frequently positive for p16 (28.6%; 12/42) than tumours in stage IV (13.1%; 11/84).

**Conclusion:**

Expression of p53, EGFR, p16 and Cyclin-D1 in non-oropharyngeal HNSCC in Ghana is largely similar to what has been reported in published studies from other countries.

## Introduction

Globally, head and neck cancers (HNCs) are among the top ten malignancies [1]. HNCs include carcinomas of the larynx, oral cavity, nasopharynx and sinonasal tract [2] and majority of HNCs are squamous cell carcinomas (SCC). The incidence of head and neck squamous cell carcinoma (HNSCC) differ widely in Africa, from 0.8 per 100,000 in Ghana to 11.1 per 100,000 in South Africa [3, 4]. Moreover, previous studies in Ghana have also shown that tumours of the pharynx, larynx and oral cavity formed the largest group of HNCs and most of the patients presented with late (stage IV) disease [5, 6]. Molecular targeted therapy seeks to block tumour growth and spread by interfering with molecular carcinogenic pathways [7]. Numerous genes including tumour suppressors like cyclin-dependent kinase inhibitors, TP53 and oncogenes like cyclin family, epidermal growth factor receptor (EGFR) and ras genes have been implicated as playing a role in HNCs [8, 9].

Mutation in p53 gene (TP53) accounts for 45–70% of HNSCC, and strategies targeting TP53 gene and p53 protein may stop or reverse the process of tumorigenesis [10]. The predictive value of Human papilloma virus (HPV) in non-oropharynx HNCs is principally unstudied, possible due to the far lower detection rates of HPV-positive tumours at these sites [11]. Higher expression of EGFR in oral squamous cell carcinomas have also been correlated with larger tumours and late stage, and hence, a poor prognosis [12, 13]. In laryngeal squamous cell carcinoma, an association of Cyclin-D1 overexpression with nodal metastases and late staged disease has been reported [14–16]. The role of the HER2 in HNCs is not well defined. Moreover, it is a proto-oncogene, with homology to the EGFR sequence and its overexpression has been observed in multiple types of cancer and used in treatment measures against cancer [17]. Based on molecular studies, molecular targeted therapies are advancing globally which seeks to block tumour growth and spread by interfering with molecular carcinogenic pathways in head and neck cancer. However, studies on molecular expression of these prognostic tumour markers in Ghana are scarce. To the author best knowledge, there is no published study on expression of molecular markers in head and neck cancers among Ghanaians. Due to the lack of molecular studies, tumour stage, patient's age and performance status still remain the basis for treatments decisions. Therefore, identification of appropriate markers that could be employed as prognostic indices of the disease, and the identification of potential candidates for molecular targeted therapy would aid in developing more suitable and effective treatment strategies for non-oropharyngeal squamous cell carcinoma. The objective of this study is to investigate the expression of p53, p16, EGFR and Cyclin-D1 among patients with non-oropharyngeal squamous cell carcinoma (HNSCC).

## Materials and methods

### Study design and setting

The study was a retrospective case series using archival pathological specimens to determine the molecular characteristics of histologically confirmed invasive SCC of non-oropharyngeal (oral cavity, larynx, sinonasal tract and nasopharynx) tissue. It was conducted at the Komfo Anokye Teaching Hospital (KATH) and KNUST, Kumasi, Ghana. The KATH Oncology Center is the second of only two national cancer centres in Ghana and the referral destination of all cancer cases seen at the hospital. Generally, cancer patients are referred for neo-adjuvant chemotherapy, adjuvant chemotherapy, concurrent chemo-radiation or radiotherapy. After treatment, all patients are followed up at the centre. The cancer centre has a clinic-based registry, which started in 2004. On the average about 400 cancer cases are seen every year.

### Ethical issues

The study protocol was registered by the Research and Development unit of the Komfo Anokye Teaching Hospital, Kumasi *(RD/CR12/130)* and approved by the Committee on Human Research, Publication and Ethics of the School of Medical Sciences, Kwame Nkrumah University of Science and Technology, Kumasi *(CHRPE/AP/353/14)*. Participants provided informed consent and their anonymity was preserved. During the whole project period, each patient was encrypted and given an ID-number. After completion of the study, all patient information collected in connection with the study was stored in the Kwame Nkrumah University of Science and Technology archives.

### Sampling population and size

A convenience sampling of 154 formalin-fixed paraffin-embedded blocks were retrieved from hospital archived pathological specimens and these specimens were collected before treatment. Case subjects were consecutive patients who had been diagnosed with HNSCC at Eye, ENT, and Oral Maxillofacial clinics of the Komfo Anokye Teaching Hospital from June 2006 to May 2014 and received treatment at the Oncology Centre of the hospital.

### Data collection tools

Data were collected using a pre-formatted data collection form. For each case, the corresponding information on age, gender, tumour size, histological type, tumour grade, tumour stage, nodes and metastases were collected from clinical record folders. The clinico-pathological features were classified according to the TNM system [18]

Two Pathologist reviewed the histology of all cases of HNSCC used in this study. High-throughput molecular pathology was don using a constructed tissue microarrays (TMAs). The most representative areas of viable tumour cells (most aggressive invasive front) were carefully selected and marked on the hematoxylin and eosin (HE) slides for the matching donor blocks and sampled for the tissue microarray collector blocks. The TMAs were assembled using a tissue-arraying instrument (Beecher Instruments). Briefly, a 0.6mm-diameter needle was used to harvest the marked tissue areas from the corresponding formalin-fixed, paraffin-embedded tissue blocks. We used two 0.6-mm cores of viable tumour tissue each from different areas that were most representative, after all original sections of the tumour have been reviewed and taken into consideration the heterogeneity of the tumour. Twelve (12) array blocks were constructed to include all core samples. This is because single section might not be enough representation of the expression of a marker throughout the whole tumour tissue, as tumour cells are regarded as heterogeneous. Multiple 4-μm sections were cut with a Micron microtome (HM355S) and stained with specific antibodies for immunohistochemistry (IHC) [19–21].

### Deparaffinizing and rehydrating

Slides were pre-treated on a heat block for 15–20 seconds. Owing to the translational nature of this study, only manual IHC staining procedures were followed as much as possible, and performed on standard whole tissue sections from paraffin-embedded archival tissue. In order to remove the paraffin wax from the tissue and allow water-soluble dyes to penetrate the sections, all slides were deparaffinized and rehydrated in graded xylene/ethanol baths before differential antigen retrieval. Briefly, all slides were washed in three changes of xylene for 3 minutes before absolute ethanol and in two changes of ethanol for 3 minutes with concentrations 95% and 70% respectively. Slides were then rinsed under running water and rinsed in phosphate-buffered saline (PBS) for 2x5 minutes. Slides were held in PBS until ready to perform antigen retrieval to prevent dehydration.

### Antigen retrieval, enzyme blocking and staining

Heat-induced epitope retrieval was performed. All slides and retrieval buffer (Citrate Buffer pH 6.0) were held in Coplin jars in a water bath set to 92°C and incubated for 25 minutes. Slides were then taken out of jars and allowed to cool on the bench for 20 minutes before washing in PBS for another 5 minutes.

An in-house staining protocol, which has been optimized for each of the antibodies, was used as described. To avoid tissue drying leading to non-specific binding and ultimately high background staining, all incubations were done out in a humidified chamber. Sections were washed in peroxidase blocking solution (3% hydrogen peroxide/casein in PBS) at room temperature for 10 minutes to block off endogenous peroxidases and rinsed in PBS for 5 minutes with gentle agitation prior to primary antibody incubation to prevent tissue destruction and to eliminate false-positive reactions produced in this way. Afterwards slides were washed in serum blocking solution (10% horse serum in casein) for 30 minutes at room temperature and washed with in PBS solution. Usually, no serum blocking is needed if antibody diluent (eg. casein) is used. This to effectively minimize non-specific staining as the protein (casein) used as a block cannot be recognized by any of the subsequent antibodies and can saturate and neutralize charged sites thus enabling the primary antibody to bind to the antigenic site only. Sections were then incubated in primary antibody at appropriate dilution in PBS with casein for 1 hour at room temperature or overnight. Sections were then rinsed in PBS for 3x5 min. Hereafter, sections were incubated in biotinylated secondary antibody in PBS for 30 minutes at room temperature and then rinsed in PBS for 3x5 min. Next, sections were incubated in ABC-Peroxidase solution for 30 minutes at room temperature and then rinsed in PBS for 3x5 min. Further, sections were incubated in peroxidase substrate solution (DAB (Sigma-Aldrich, Darmstadt, Germany) for 30 minutes at room temperature and then rinsed in double-distilled water for 5 min. A counterstain, hemotoxylin was then applied to all sections and rinsed in double-distilled water for 2–5 minutes. Finally, the sections were dehydrated by washing in 95% ethanol for 1 minute, 100% ethanol for 2x3min, cleared in xylene for 2x5min and coverslip with mounting medium. A negative control where the primary antibody was omitted was included for all antibodies used and showed no staining in all cases of IHC staining [22].

### Scoring

All stained sections were examined by the pathologists without knowledge of clinical outcome. The two blinded pathologists scored independently and semi-quantitatively viable parts of each anonymized core by light microscopy. The extent and location of immunohistochemical staining for individual molecular markers were assessed using the immunoreactive score (IRS) to evaluate the proportion of cells expressing molecular marker of interest and the intensity of staining. The scoring was semi-quantitative according to extensively used protocols [23, 24]. Percentage of the cancer area stained in high-power fields was examined. Staining intensity of epithelial tumour cells was scored as follows: 0 (negative); 1 (weak); 2 (intermediate); and 3 (strong). Percentage of positive cells examined was scored as 0 (negative), 1 (< 10%), 2 (11–50%), 3 (51–80%), and 4 (> 80%). The two scores were multiplied and the IRS (values from 0–12) was determined: 0–3 as negative, values 4–6 as positive, and multiplication values 8, 9, 12 as strongly positive [25]. A core was scored as missing if the core was missing or considered to be of inadequate quality to score by both observers. Consequently, all reported marker expression were based on the evaluation of three distinct tissue cores [26, 27].

### Statistical analysis

Data were entered and analysed using SPSS. Exploratory analyses was done to obtain descriptive statistics. Correlations between the biological markers and the different categorical variables were assessed with Pearson`s chi-square test. Associations with p<0.05 were considered significant.

## Results

The age of patients ranged from 15–98 years with mean 59.1 years. One hundred and eight (70.1%) of the patients were males. The commonest primary site was the larynx (41.3%) followed by oral cavity (36.1%). The histological grade of the tumours was mostly moderately differentiated or poorly differentiated grade. More than half of the patients (66.2%) had clinical stage IV and 38.3% had no nodal involvement. Twelve had distant metastases [Table 1].

**Table 1:** Age, gender and clinico-pathological characteristics of the patients (n = 154).

Variables	Frequency (n)	Percentages (%)
Age (years) (Mean ± SD)	59.1±16.3	
**Age Groups (years)**		
<40	22	14.3%
41–60	56	36.4%
>60	76	49.3%
**Gender**		
Male	108	70.1%
Female	46	29.9%
**Clinical Stage**		
I	3	2.0%
II	16	10.4%
III	33	21.4%
IV	102	66.2%
**Histological Grade**		
Well differentiated (G1)	17	11.0%
Moderately differentiated (G2)	74	48.1%
Poorly differentiated (G3)	63	40.9%
**Tumor grade**		
T1	6	3.9%
T2	41	26.6%
T3	38	24.8%
T4	69	44.8%
**Nodal Involvement**		
N0	59	38.3%
N1	29	18.8%
N2	42	27.3%
N3	24	15.6%
**Metastasis**		
M0	142	92.2%
M1	12	7.8%
**Tumor Site**		
Nasal	34	22.1%
Larynx	64	41.6%
Oral Cavity	56	36.3%

SD = Standard Deviation

Table 2 shows the frequency distribution of expression of the molecular markers. Testing was unsuccessful in 18.2 to 43.5 percent of all cases. In the remaining cases, moderately or strongly positive expression varied from 1% for HER2, 10% for Cyclin-D1 and 18.3% for p16 to 48.3% for EGFR.

**Table 2:** Frequency distribution of molecular markers expression of non-oropharyngeal HNSCC patients (n = 154).

Variables	Frequency (n)	Percentages (%)
**p16**		
Positive	23	18.3%
Negative	103	81.7%
Invalid Results	28	
**p53**		
Positive	29	24.0%
Negative	92	76.0%
Invalid Results	33	
**EGFR**		
Strongly positive	25	28.7%
Moderately Positive	17	19.5%
Negative	45	51.7%
Invalid Results	67	
**HER2**		
Strongly positive	0	0.0%
Moderately Positive	1	1.0%
Negative	96	99.0%
Invalid Results	57	
**Cyclin-D1**		
Positive	12	10.0%
Negative	108	90.0%
Invalid Results	34	

**Fig 1:**
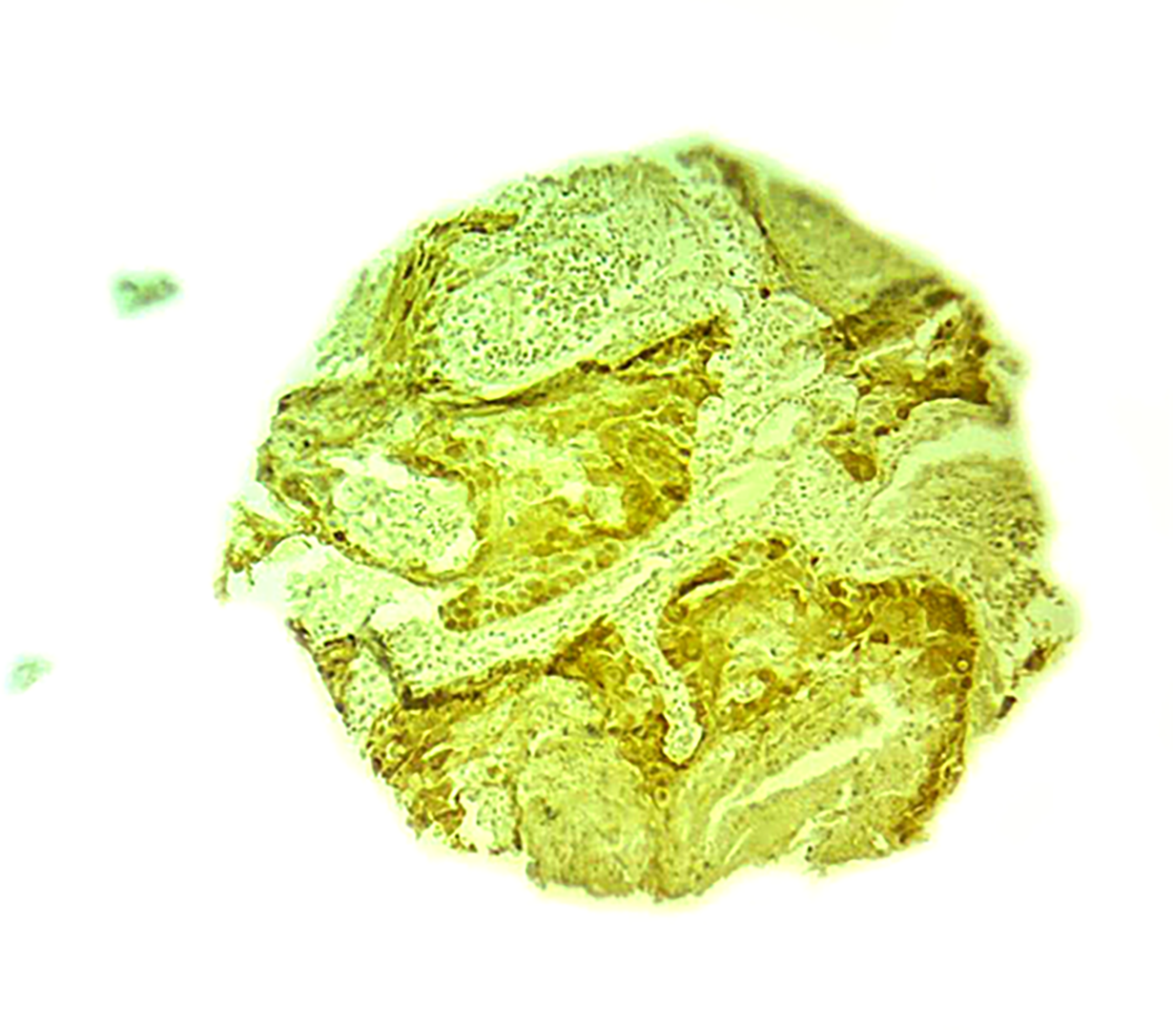
Microphotograph of the immunohistochemistry for p16 positive. Fig 1 shows microphotograph of the immunohistochemistry for positive expression of p16.

**Fig 2:**
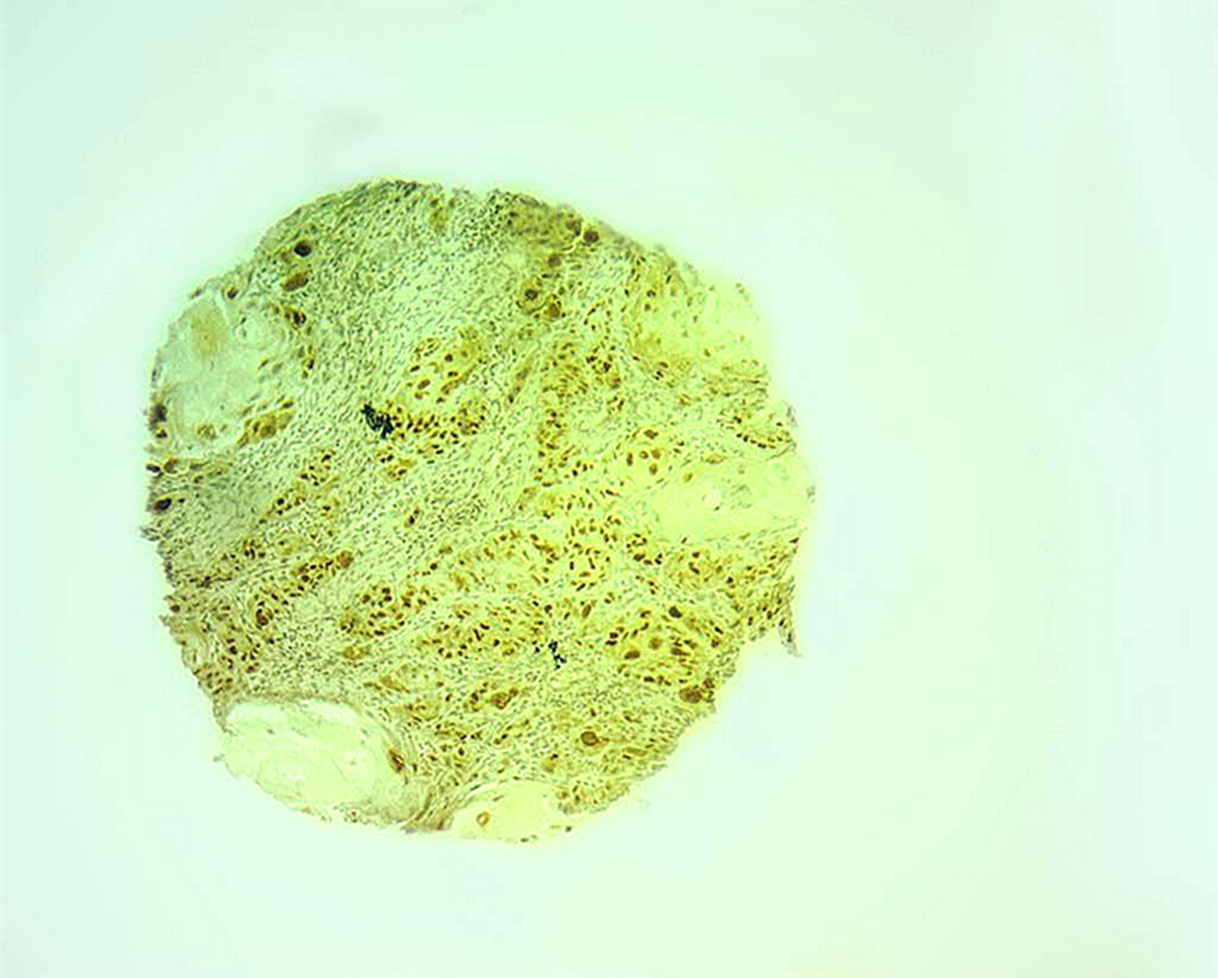
Microphotograph of the immunohistochemistry for p53 positive. Fig 2 shows the microphotograph of the immunohistochemistry for positive expression of p53.

Figs 1 and 2 shows the micrographs of the immunochemistry for the positive expression of p16 and p53.

Among the 87 patients that were successfully tested, (42; 48.3%) showed strong or moderate expression of EGFR. There was no statistically significant association of EGFR expression with age, gender, or clinico-pathological features. There was a significantly (p = 0.022) higher expression of Cyclin-D1 in tumours of the oral cavity (19.6%) than in those of the larynx (4.7%) and nose (3.2%), but no significant correlation of expression of Cyclin-D1 expression with age, gender, or clinico-pathological features was noted. Tumours in stages I-III were more frequently positive for p16 (28.6%) than tumours in stage IV (13.1%), but the difference was only of borderline statistical significance (p = 0.049). There was no statistically significant association of p16 expression with age, gender, or clinico-pathological features. No significant association between p53 expression and age, gender, or clinico-pathological features was observed **[Table 3].**

**Table 3:** Association between age, gender, and clinico-pathological features and the expression of molecular markers among non-oropharyngeal HNSCC patients.

	EGFR		Cyclin-D1		p16		p53	
Variables	n	Positive (%)	n	Positive (%)	n	Positive (%)	n	Positive (%)
**Age (years)**								
≤ 60	40	21 (52.5%)	65	7 (10.8%)	66	12 18.2%)	65	14 (21.5%)
> 60	47	21 (44.7%)	55	5 (9.1%)	60	11 (18.3%)	56	15 (26.8%)
**Gender**								
Male	64	31 (48.4%)	82	10 (12.2%)	86	17 (19.8%)	82	21 (25.6%)
Female	23	11 (47.8%)	38	2 (5.3%)	40	6 (15.0%)	39	8 (20.8%)
**Tumour site**								
Nasal	22	8 (36.4%)	31	1 (3.2%) *	31	3 (9.7%)	30	8 (26.7%)
Larynx	37	20 (54.1%)	43	2 (4.7%)	51	12 (23.5%)	44	9 (20.5%)
Oral cavity	28	28 (50.0%)	46	9 (19.6%)	44	8 (18.2%)	47	12 (25.5%)
**Tumour Stage**								
I-III	31	14 (45.2%)	41	5 (12.2%)	42	12 (28.6%) *	41	10 (24.4%)
IV	56	28 (50.0%)	79	7 (8.9%)	84	11 (13.1%)	80	19 (23.8%)
**Tumour grade**								
G3	41	18 (43.9%)	48	4 (8.3%)	50	7 (14.0%)	51	10 (19.6%)
G2	35	19 (54.3%)	59	7 (11.9%)	62	15 (24.2%)	57	15 (26.3%)
G1	11	5 (45.5%)	13	1 (7.7%)	14	1 (7.1%)	13	4 (30.8%)
**Tumour size**								
T1-T3	49	25 (51.0%)	67	6 (9.0%)	70	17 (24.3%)	65	14 (21.5%)
T4	38	17 (44.7%)	53	6 (1.3%)	56	6 (10.7%)	56	15 (26.8%)
**Nodal involvement**								
N0/N1	49	19 (38.8%)	79	7 (8.9%)	72	15 (20.8%)	69	14 (20.3%)
N2/N3	38	23 (60.5%)	41	5 (12.2%)	54	8 (9.3%)	52	15 (28.8%)
**Metastasis**								
M0	77	38 (49.4%)	111	11 (9.9%)	116	22 (18.9%)	112	28 (25.0%)
M1	10	4 (40.0%)	9	1 (11.1%)	10	1 (10.0%)	9	1 (11.1%)

n = number of successfully tested patients

* p < 0.05

As shown in Table 4, among the 72 successful tested cases, (11; 15.1%) showed positive co-expression of EGFR+p53, 8 (11.4%) showed two-marker positive co-expression (EGFR+P16), 6 (8.5%) also showed two-marker co-expression of EGFR+Cyclin-D1 and 3 (4.7%) co-expression of all three markers (EGFR+p53+p16).

**Table 4:** Combined positivity of EGFR and other markers.

Marker	Successfully tested cases	Positive (%)
EGFR	87	42 (48.3%)
EGFR + p53	72	11 (15.1%)
EGFR + p16	70	8 (11.4%)
EGFR + p53 + p16	64	3 (4.7%)
EGFR + Cyclin–D1	71	6 (8.5%)
EGFR + p53 + p16 + Cyclin–D1	60	-

## Discussion

Head and neck squamous cell carcinomas (HNSCCs) are thought to be developed and to progress through a series of genetic alterations, such as those comprising TP53 and CDKN2A. HNSCCs also show numerous abnormalities in the chromosomes including amplifications of region 11q13, which contains the cyclin-D1 gene, and region 7p11, which encodes epidermal growth factor receptor (EGFR) [28].

In our study, EGFR was the most expressed molecular marker (29.4%) followed by p53 (24.0%), p16 (18.3%) and Cyclin-D1 (10.0%) respectively. Cyclin-D1 was significantly expressed in tumours of the oral cavity (19.6%) compared to those of the larynx (4.7%) and nose (3.2%). Positive expression of p16 (28.6%) was higher in stages I-III tumours compared to tumours in stage IV (13.1%). Non-oropharyngeal HNSCC in our study consisted of cancers of the larynx, sinonasal tract, oral cavity and nasopharynx. About half of all patients were at least 60 years and 70.3% were males. In a study by Rivera et al., [29] among Chileans, the mean age was in the seventh decade for patients with oral cavity squamous cell carcinoma (OCSCC) and predominantly affected males which is similar to our current findings and other previous reports for OCSCC from Sri Lanka and Brazil [30, 31]. The results of the present study also showed that more than half of the patients had clinical stage IV which is consistent with studies by Rivera et al., [29] and Rodrigues et al., from Brazil.

EGFR was positive in 48.3%of the successful tested patients in this study. This finding is similar to report from study done by Ang et al who demonstrated that increased aggressiveness of the collective group of tumours with expression scores above the median (50%). Dragomir et al., reported that EGFR expression did not correlate with the degree of differentiation of the OCSCC, but was higher expressed in well and moderately differentiated tumours which is consistent with our findings [32]. The importance of EGFR as a high-risk indicator for progressive disease or poor prognosis has been demonstrated in head and neck cancers [33, 34].

The result of the study found p53 expression in 24.4% of non-oropharyngeal HNSCC, which is lower than 64% reported Nylander et al., [35] but similar to findings from previous studies [36, 37]. Shiraki et al., [38] observed statistically insignificant association between Cyclin-D1 or p53 expression and any clinico-pathological characteristics in the oral cancer patient. In patients with LSCC, p53 expression does not seem to be associated with biological features. [39] and this confirms findings from our present study.

Protein expression levels of Cyclin-D1 are related to the site of the tumour, depth of tumour invasion and stage of the disease.[40, 41]. In addition, Krecicki et al., [42] found that Cyclin-D1 was principally overexpressed in locally advanced tumours (T3–T4). In other studies, correlation was detected between overexpression of Cyclin-D1 and advanced stage of disease and nodal metastases [14–16]. With the exception of tumour site, no association was observed with nodal metastases and Cyclin-D1 expression in our current study. We found expression of Cyclin-D1 in 10% of the patients whereas other studies reported higher expression [14, 43].

Shiraki et al., indicated that it is only the combination of positive expression of EGFR, p53, and Cyclin-D1 that predicts poor prognosis [29]. The results found that positive co-expression of all three markers (EGFR+p53+p16) was observed in 3 (4.7%). Such co-expression of many molecular markers agrees relatively well with the fundamental concept of multistep development and progression of cancer [29].

This study has a relatively large proportion of missing cases due to invalid results of the immunohistochemical staining, largely because of missing tissue in the TMA or on the slide. Despite using plus glass, sections from the small cores in the TMA may loosen from the slide in the process of immunohistochemical staining. However, a comparison of the distributions of age, gender, and clinico-pathological variables of the total material in Table 1 with those of the successfully tested cases in Table 3 show no obvious significant difference, indicating that the successfully tested cases may be a reasonably representative sample of the total material.

Two of the comparisons shown in Table 3 showed differences that were statistically significant at the defined level of p<0.05; the difference in Cyclin-D1 expression in tumours of different sites (p = 0.022), and the difference in p16 expression in different stages (p = 0.049). However, as in this study with over 30 statistically tested differences, this is not more than can be expected to occur by pure chance in testing this number of random samples from populations between which no real difference exists. Thus, as a whole, our study has not demonstrated significant associations between the expression of EGFR, Cyclin-D1, p16, or p53 and age, gender, tumour site, stage, grade, size, nodal involvement, or metastasis in patients with non-oropharyngeal HNSCC. Moreover, the study was conducted with small sample size at a single hospital; the results obtained from the study cannot be generalized to the population.

## Conclusion

Expression of p53, EGFR, p16 and Cyclin-D1 in non-oropharyngeal HNSCC in Ghana is largely similar to what has been reported in published studies from other countries. Further prospective studies are warranted to determine the outcome of expression of these markers on the overall survival in non-oropharyngeal HNSCC patients.

## Supporting information

S1 Dataset Dataset on which conclusions of this manuscript were made has been submitted as supporting information. (XLSX)
