# Correction: Expression of immunohistochemical markers in non-oropharyngeal head and neck squamous cell carcinoma in Ghana

**DOI:** 10.1371/journal.pone.0209696

**Published:** 2018-12-19

**Authors:** Osei Owusu-Afriyie, W. K. B. A. Owiredu, Kwabena Owusu-Danquah, Christine Komarck, Susan K. Foltin, Rita Larsen-Reindorf, Emmanuel Acheampong, Solomon E. Quayson, Mark E. Prince, Jonathan B. McHugh, Peter Donkor, Sofia D. Merajver, J. Chad Brenner

There are authors who should be included in the author byline. Christine Komarck should be listed as the fourth author, and the contributions of this author are as follows: Data acquisition. Susan K. Foltin should be listed as the fifth author, and the contributions of this author are as follows: Data acquisition. Mark E. Prince should be listed as the ninth author, and the contributions of this author are as follows: Resources, Supervision. Jonathan B. McHugh should be listed as the tenth author, and the contributions of this author are as follows: Data analysis and interpretation, Pathology oversight. Sofia D. Merajver should be listed as the 12th author, and the contributions of this author are as follows: Resources, Funding acquisition, Supervision, Conception, Data interpretation. J. Chad Brenner should be listed as the 13th author, and the contributions of this author are as follows: Resources, Funding acquisition, Supervision, Conception, Data interpretation, Overall project oversight.

Please view the complete and correct author byline, affiliations, and citation here:

Osei Owusu-Afriyie^1,2^, W. K. B. A. Owiredu^1^, Kwabena Owusu-Danquah^3^, Christine Komarck^4^, Susan K. Foltin^4^, Rita Larsen-Reindorf^5^, Emmanuel Acheampong^2^, Solomon E. Quayson^6^, Mark E. Prince^4,7^, Jonathan B. McHugh^8,9^, Peter Donkor^5,10^, Sofia D. Merajver^11,^*, and J. Chad Brenner^4,12,^*

**1** Department of Molecular Medicine, School of Medical Sciences, Kwame Nkrumah University of Science and Technology (KNUST), Kumasi Ghana, **2** Department of Pathology, Komfo Anokye Teaching Hospital, Kumasi Ghana, **3** Department of Medical Laboratory Technology, Faculty of Allied Health, Kwame Nkrumah University of Science and Technology (KNUST), Kumasi Ghana, **4** Department of Otolaryngology, Head and Neck Surgery, Rogel Cancer Center, University of Michigan Medical School, **5** Directorate of Dental, Eye, Ear, Nose & Throat, Komfo Anokye Teaching Hospital (KATH), Kumasi, Ghana, **6** Department of Pathology, University of Ghana Medical School, Accra Ghana, **7** University of Michigan Medical School, Rogel Cancer Center, University of Michigan Medical School, **8** Department of Pathology, Rogel Cancer Center, University of Michigan Medical School, **9** Department of Oral and Maxillofacial Surgery, Rogel Cancer Center, University of Michigan Medical School, **10** Department of Maxillofacial Surgery, Dental School, KNUST, **11** Department of Internal Medicine (Division of Hematology-Oncology), University of Michigan, Rogel Cancer Center, University of Michigan, **12** Department of Pharmacology, Rogel Cancer Center, University of Michigan Medical School.

Owusu-Afriyie O, Owiredu WKBA, Owusu-Danquah K, Komarck C, Foltin SK, Larsen-Reindorf R, et al. (2018) Expression of immunohistochemical markers in non-oropharyngeal head and neck squamous cell carcinoma in Ghana. PLoS ONE 13(8): e0202790. https://doi.org/10.1371/journal.pone.0202790.

The following information is missing from the Funding section: This study was supported by NIH grant NCI:P30:CA046592-S1 to investigators Dr. Merajver and Dr. Brenner, the University of Michigan Rogel Cancer Center, and the University of Michigan African Studies Center.

The following information is missing from the Materials and methods section: The study protocol was registered by the Research and Development unit of the Komfo Anokye Teaching Hospital, Kumasi (RD/CR12/130) and approved by the Committee on Human Research, Publication and Ethics of the School of Medical Sciences, Kwame Nkrumah University of Science and Technology, Kumasi (CHRPE/AP/353/14). During the whole project period, each patient was encrypted and given an ID-number. After completion of the study, all patient information collected in connection with the study was stored in the Kwame Nkrumah University of Science and Technology archives. Additional ethics board approval was obtained at the University of Michigan for the molecular analyses of tissue blocks (HUM00098456). Tissue microarray construction was completed at the University of Michigan Rogel cancer center histology core facility.
